# Supplementary material for: A Deeper Investigation of Drug Degradation Mixtures Using a Combination of MS and NMR Data: Application to Indapamide
Source: Molecules. 2019 May 7;24(9):1764. doi: 10.3390/molecules24091764 (PMC6539681; doi:10.3390/molecules24091764)
Supplement: Supplementary file 1 [file molecules-24-01764-s001.pdf]

A deeper investigation of drug degradation mixtures using a combination of MS and NMR data: application to indapamide

Cécile Palaric [1,2], Roland Molinié [2], Dominique Cailleu [1], Jean-Xavier Fontaine [2], David Mathiron [1], François Mesnard [2], Yoann Gut [3], Tristan Renaud [3], Alain Petit [3] and Serge Pilard [1,*]

SUPPLEMENTARY MATERIALS

Table S1

Methods for the determination of detection and quantification limits of API for NMR and UV/MS experiments and their respective calculated values.

Calibration curves provided linear equations: $y = a_0 + a_1x$, where y is the response, x the API concentration, a_1 the slope coefficient and a_0 the intercept coefficient corresponding to noise.

$$\mathrm{LOD} = 3.3 \times \frac{\text{Standard deviation of the intercept } (S_{a_0})}{\text{Slope of linear equation } (a_1)}$$

$$\mathrm{LOQ} = 10 \times \frac{\text{Standard deviation of the intercept } (S_{a_0})}{\text{Slope of linear equation } (a_1)}$$

We calculated the lack of fit of the linear model using the R software and more particularly thanks to the pure.error.anova function of the alr3 pakage. This test is based on an F-test (with $\alpha = 0.05$): under the H_0 hypothesis: there is no lack of fit in the linear regression model while under the H_1 hypothesis: there is a lack of fit in the linear regression model.

	**Slope (± SD)**	**Intercept (± SD)**	**R^2**	**p-value (F-test)**	**LOD (mM)**	**LOQ (mM)**
**UV**	3.43E6±3.66 E4	-3.08E3±1.12E3	0.9961	0.4135	0.001	0.003
**ESI^+ MS**	2.83E6±3.32E4	-1.94±1.02E3	0.9961	0.8608	0.001	0.004
**ESI^- MS**	7.39E6±7.82E4	-1.75E3±7.39E4	0.9969	0.5840	0.001	0.003
**^1H NMR**	1.84E-2±1.15E-4	-2.21E-3±6.99E-4	0.9989	0.2481	0.125	0.380

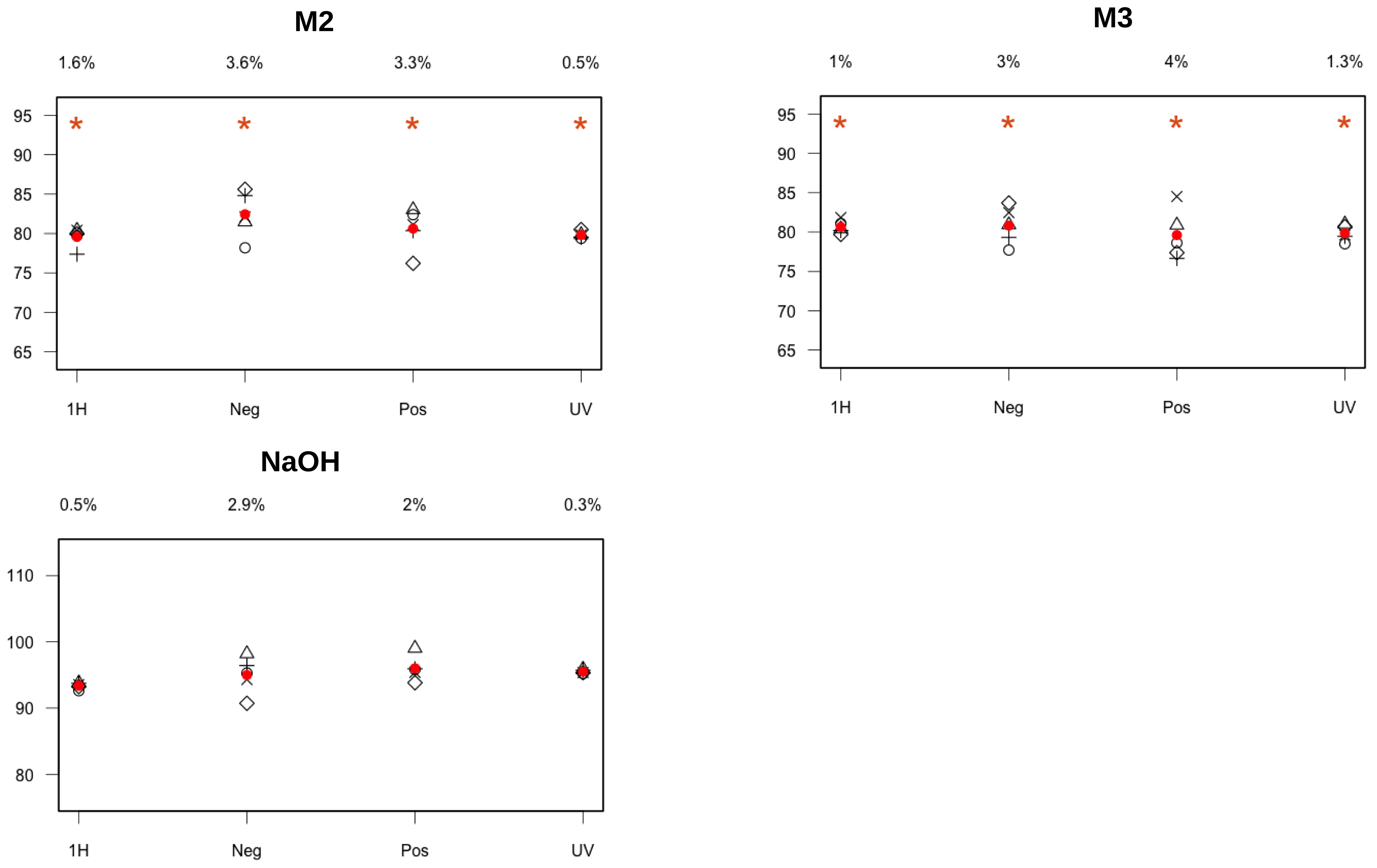

Figure S1. Quantification of API (%; Y axis) for M2, M3 and NaOH degradation with different methods (^{1}H NMR, ESI: negative and positive ion mode, UV at 275 nm; X axis) and for the 5 replicates (×, ◊, ○, +, Δ with ● represents the mean value). The coefficient of variation (CV) for each technique is indicated (top) and the significant of result is described by * for M2, M3 where theoretical values are available. NB: API degradation with Cu(II) is total.

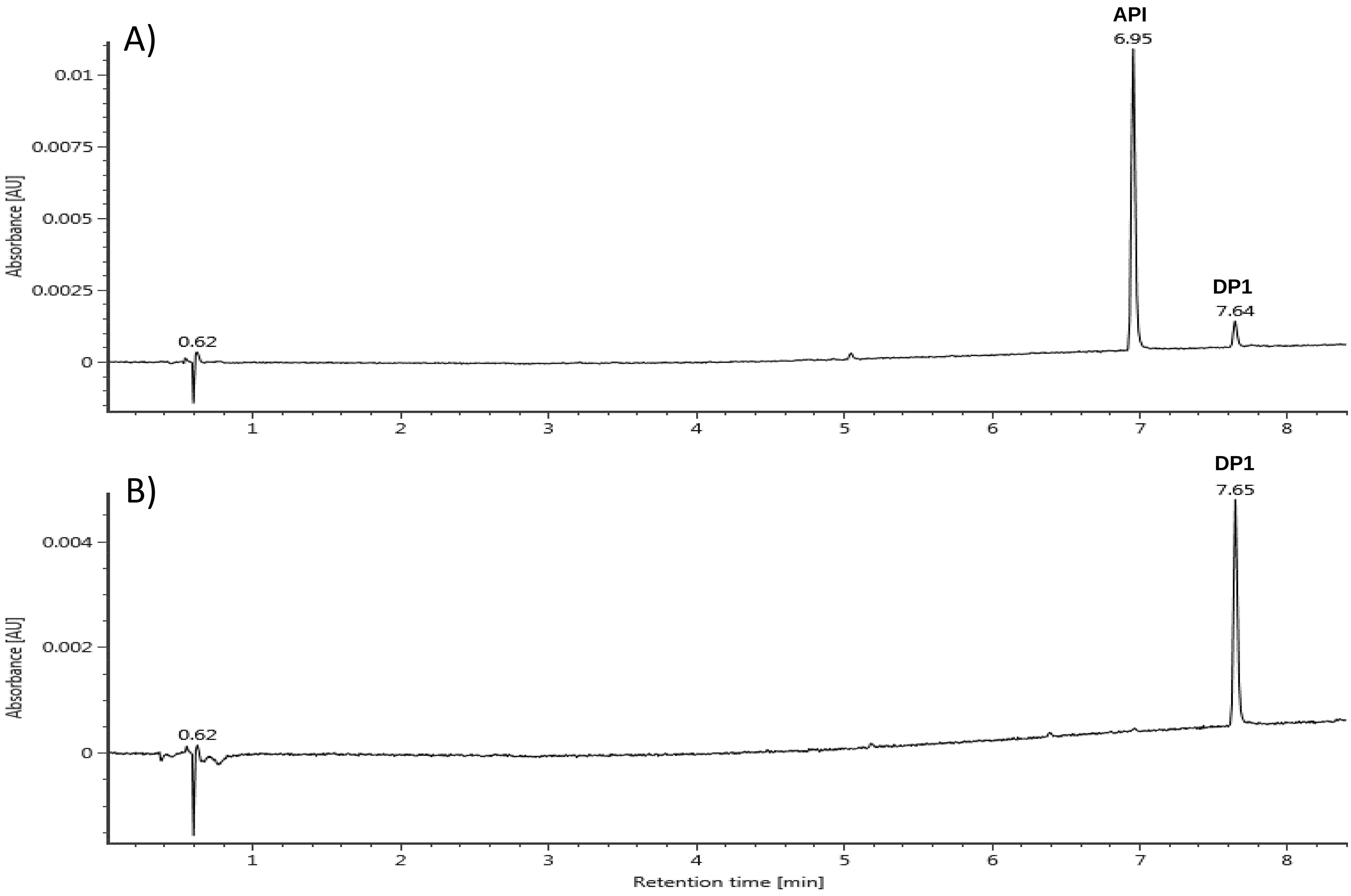

Figure S2. UV chromatograms of NaOH (A) and Cu(II) (B) degradations.

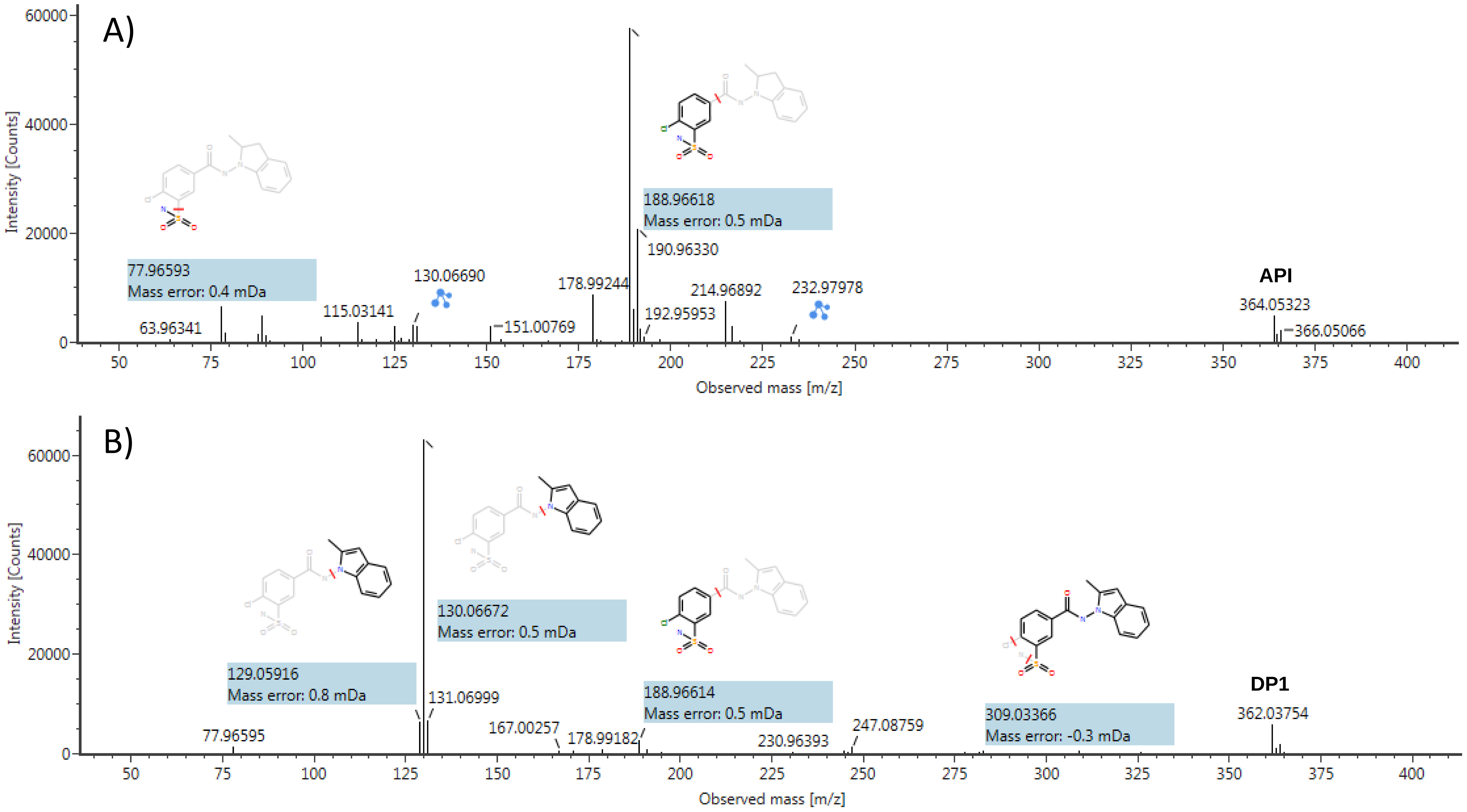

Figure S3. Negative HDMS^E spectra of API (A) and DP1 (B).

Table S2

Structure hypotheses for DP1 and DP3 and predicted $^{1}H/^{13}C$ NMR data (MNova).

**Compounds**	**Positions**	**δC (ppm)**	**δH (ppm)**	**Multiplicity, *J*(Hz), nH**
DP3	Tr (min) = 3.22			
4-chloro-3-sulfamoylbenzoic acid 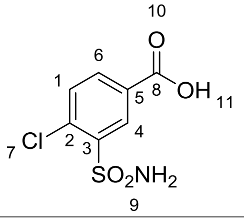	1	128.76	7.95	d, *J* = 8.4 Hz, 1H
	2	129.77	-	-
	3	136.57	-	-
	4	129.73	8.47	d, *J* = 1.9 Hz, 1H
	5	128.07	-	-
	6	131.28	8.39	dd, *J* = 8.3, 1.9 Hz, 1H
	8	166.27	-	-
DP1	Tr (min) = 7.67			
4-chloro-N-(2-methyl-1H-indol-1-yl)-3-sulfamoylbenzamide 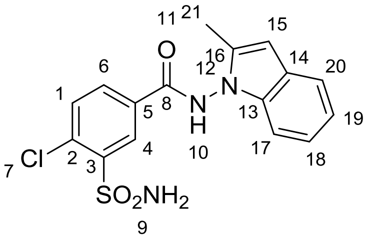	1	131.09	7.92	d, *J* = 8.2 Hz, 1H
	2	130.60	-	-
	3	137.29	-	-
	4	126.11	8.62	d, *J* = 1.9 Hz, 1H
	5	131.91	-	-
	6	130.71	8.26	dd, *J* = 8.2, 1.9 Hz, 1H
	8	163.36	-	-
	13	140.32	-	-
	14	128.43	-	-
	15	102.53	6.34	dh, *J* = 2.0, 0.5 Hz, 1H
	16	142.36	-	-
	17	111.61	7.50	ddt, *J* = 6.2, 1.7, 0.5 Hz, 1H
	18	124.35	7.05	ddd, *J* = 6.9, 6.2, 1.2 Hz, 1H
	19	120.63	7.11	dddd, *J* = 7.4, 6.9, 1.6, 0.5 Hz, 1H
	20	121.21	7.22	dddd, *J* = 7.4, 1.9, 1.2, 0.5 Hz, 1H
	21	11.61	2.29	d, *J* = 0.5 Hz, 3H
4-chloro-N-(2-methyleneindolin-1-yl)-3-sulfamoylbenzamide 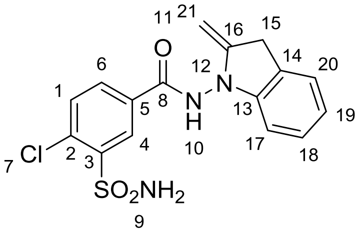	1	131.09	7.92	d, *J* = 8.2 Hz, 1H
	2	130.60	-	-
	3	137.29	-	-
	4	126.11	8.62	d, *J* = 1.9 Hz, 1H
	5	131.91	-	-
	6	130.71	8.25	dd, *J* = 8.2, 1.9 Hz, 1H
	8	163.36	-	-
	13	141.72	-	-
	14	128.07	-	-
	15	29.12	3.22	q, *J* = 1.0 Hz, 2H
	16	150.33	-	-
	17	112.35	6.87	dd, *J* = 7.8, 1.5 Hz, 1H
	18	126.04	7.26	td, *J* = 7.7, 2.4 Hz, 1H
	19	125.02	6.59	td, *J* = 7.7, 1.5 Hz, 1H
	20	123.93	7.02	ddt, *J* = 7.7, 2.4, 1.0 Hz, 1H
	21	86.20	4.19	t, *J* = 1.0 Hz, 2H

Table S3

Structure hypotheses for DP5 and predicted $^{1}H/^{13}C$ NMR data (MNova).

**Compounds**	**Positions**	**δC (ppm)**	**δH (ppm)**	**Multiplicity, *J*(Hz), nH**
DP5	Tr (min) = 3.91			
1H-benzo[c][1,2]diazepine (benzodiazepine)	1	140.09	-	-
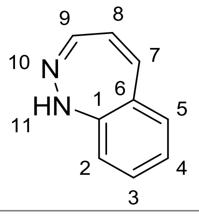	2	118.51	7.30	dd, *J* = 7.5, 1.5 Hz, 1H
	3	126.31	7.19 -7.13	m, 1H
	4	123.88	7.42	ddd, *J* = 7.8, 7.1, 1.5 Hz, 1H
	5	127.33	7.48	ddd, *J* = 7.8, 1.6, 0.6 Hz, 1H
	6	127.52	-	-
	7	130.63	6.90	ddd, *J* = 10.7, 1.3, 0.6 Hz, 1H
	8	127.72	5.30	dd, *J* = 10.7, 8.9 Hz, 1H
	9	144.17	7.94	dd, *J* = 8.9, 1.3 Hz, 1H
5-phenyl-1H-pyrazole	1	128.88	7.85-7.81	m, 1H
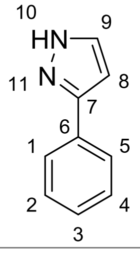	2	127.83	7.47-7.39	m, 1H
	3	129.85	7.47-7.39	m, 1H
	4	127.83	7.47-7.39	m, 1H
	5	128.88	7.85-7.81	m, 1H
	6	131.80	-	-
	7	146.72	-	-
	8	102.45	6.53	d, *J* = 2.5 Hz, 1H
	9	131.76	7.51	d, *J* = 2.5 Hz, 1H
3-methylcinnoline	3	155.24	-	-
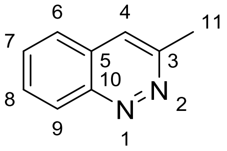	4	121.95	7.49	dh, *J* = 2.1, 0.5 Hz, 1H
	5	130.07	-	-
	6	128.34	7.22	dddd, *J* = 8.5, 2.2, 1.2, 0.5 Hz, 1H
	7	129.55	7.72	dddd, *J* = 8.4, 7.8, 1.2, 0.5 Hz, 1H
	8	129.39	7.37	ddd, *J* = 8.3, 7.8, 1.1 Hz, 1H
	9	128.81	8.05	ddt, *J* = 8.4, 1.1, 0.5 Hz, 1H
	10	148.30	-	-
	11	21.80	2.08	d, *J* = 0.5 Hz, 3H
4-methylcinnoline	3	145.49	8.90	p, *J* = 0.6 Hz, 1H
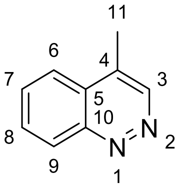	4	140.13	-	-
	5	128.54	-	-
	6	125.97	7.43-7.37	m, 1H
	7	134.41	7.81	ddd, *J* = 8.1, 7.0, 1.2 Hz, 1H
	8	129.10	7.43-7.37	m, 2H
	9	129.10	8.13	ddd, *J* = 8.4, 1.2, 0.6 Hz, 1H
	10	148.92	-	-
	11	18.90	2.39	d, *J* = 0.6 Hz, 3H
1-methylphtalazine	1	156.21	-	-
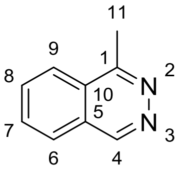	4	149.30	9.64-9.60	m, 1H
	5	124.41	-	-
	6	123.94	8.25-8.19	m, 1H
	7	126.22	8.11-8.06	m, 1H
	8	127.49	8.11-8.06	m, 1H
	9	126.72	8.25-8.19	m, 1H
	10	126.96	-	-
	11	20.07	1.93	s, 3H
4-phenyl-1-H-pyrazole	1	126.61	7.60-7.54	m, 1H
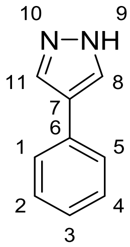	2	128.91	7.48-7.42	m, 1H
	3	127.69	7.42-7.36	m, 1H
	4	128.91	7.48-7.42	m, 1H
	5	126.61	7.60-7.54	m, 1H
	6	133.38	-	-
	7	124.95	-	-
	8	127.79	8.04	s, 1H
	11	132.51	8.04	s, 1H
3-amino-3-phenylacrylonitrile	1	127.47	7.72-7.66	m, 1H
(ß-aminocinnamonitrile)	2	128.07	7.58-7.50	m, 1H
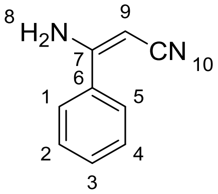	3	128.29	7.58-7.50	m, 1H
	4	128.07	7.58-7.50	m, 1H
	5	127.47	7.72-7.66	m, 1H
	6	132.62	-	-
	7	163.72	-	-
	9	58.52	4.19	s, 1H
	10	117.19	-	-

Table S4

Experimental NMR assignment of API and its degradation products (DPs) in HCl mixture. The selected proton for qNMR is described by *.

	Positions	δC (ppm)	δH (ppm)	Multiplicity, *J*(Hz), nH
**API (S1520)**	1	134.02	7.88	d, *J* = 8.3 Hz, 1H
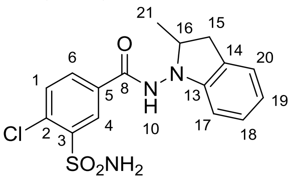	2	135.87	-	-
	3	142.37	-	-
	4	129.94	8.50	d, *J* = 2.2 Hz, 1H
	5	133.63	-	-
	6	134.03	8.16	dd, *J* = 8.3, 2.2 Hz, 1H
	8	167.19	-	-
	13	152.85	-	-
	14	129.43	-	-
	15	37.09	2.66	dd, *J* = 11.2, 15.6 Hz, 1H
	15’	37.09	3.21	dd, *J* = 8.1, 15.6 Hz, 1H
	16	-	3.95	s, 1H
	17*	110.80	6.56	d, *J* = 7.7 Hz, 1H
	18	129.06	7.12	t, *J* = 7.7 Hz, 1H
	19	122.58	6.87	t, *J* = 7.4 Hz, 1H
	20	126.57	7.20	d, *J* = 7.4 Hz, 1H
	21	19.93	1.35	d, *J* = 6.2 Hz, 3H
**DP1 (Y38)**	1	134.33	7.96	d, *J* = 8.3 Hz, 1H
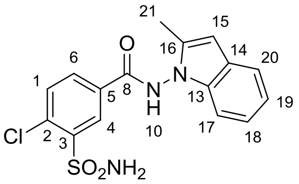	2	136.78	-	-
	3	142.64	-	-
	4*	129.04	8.61	d, *J* = 2.2 Hz, 1H
	5	132.38	-	-
	6	134.37	8.34	dd, *J* = 8.3, 2.2 Hz, 1H
	8	167.29	-	-
	13	127.60	-	-
	14	137.39	-	-
	15	100.59	6.40	t, *J* = 0.9 Hz, 1H
	16	138.95	-	-
	17	121.64	7.56	d, *J* = 7.7 Hz, 1H
	18	122.14	7.13	td, *J* = 7.1, 0.9 Hz, 1H
	19	123.25	7.18	td, *J* = 7.1, 0.9 Hz, 1H
	20	110.08	7.24	d, *J* = 8.0 Hz, 1H
	21	12.86	2.33	d, *J* = 0.9 Hz, 3H
**DP3 (Y36)**	1*	133.06	7.69	d, *J* = 8.2 Hz, 1H
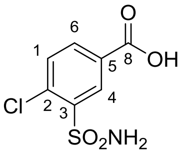	2	133.54	-	-
	3	141.27	-	-
	4	131.22	8.49	d, *J* = 2.0 Hz, 1H
	5	139.36	-	-
	6	135.61	8.07	dd, *J* = 8.2, 2.0 Hz, 1H
	8	170.88	-	-
**DP5**	3	156.00	-	-
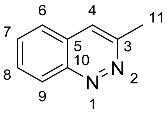	4	124.85	8.18	s, 1H
	5	133.67	-	-
	6	128.60	8.06	d, *J* = 0.9 Hz, 1H
	7	133.81	7.92	d, *J* = 0.4 Hz, 1H
	8	132.81	7.98	td, *J* = 6.7, 1.2 Hz, 1H
	9*	129.92	8.45	dd, *J* = 8.5, 0.7 Hz, 1H
	10	150.47	-	-
	11	23.02	2.92	d, *J* = 0.4 Hz, 3H

A) NMR spectrum was recorded on Bruker Avance NEO 900 MHz spectrometer with a 5-mm TCI cryoprobe. The 2D HMBC (Heteronuclear Multiple-Bond Correlation spectroscopy) spectrum was acquired with the Bruker library *hmbcetgpl3nd*, with a 2 s relaxation delay using 64 scans per 8 K increments, which were collected into 4 K data points, using spectral widths of 9090 Hz in F2 and 45276 Hz in F1. The number of NUS sampling points was 256 complex points (3.125 % sampling density of 8 K points).

B)

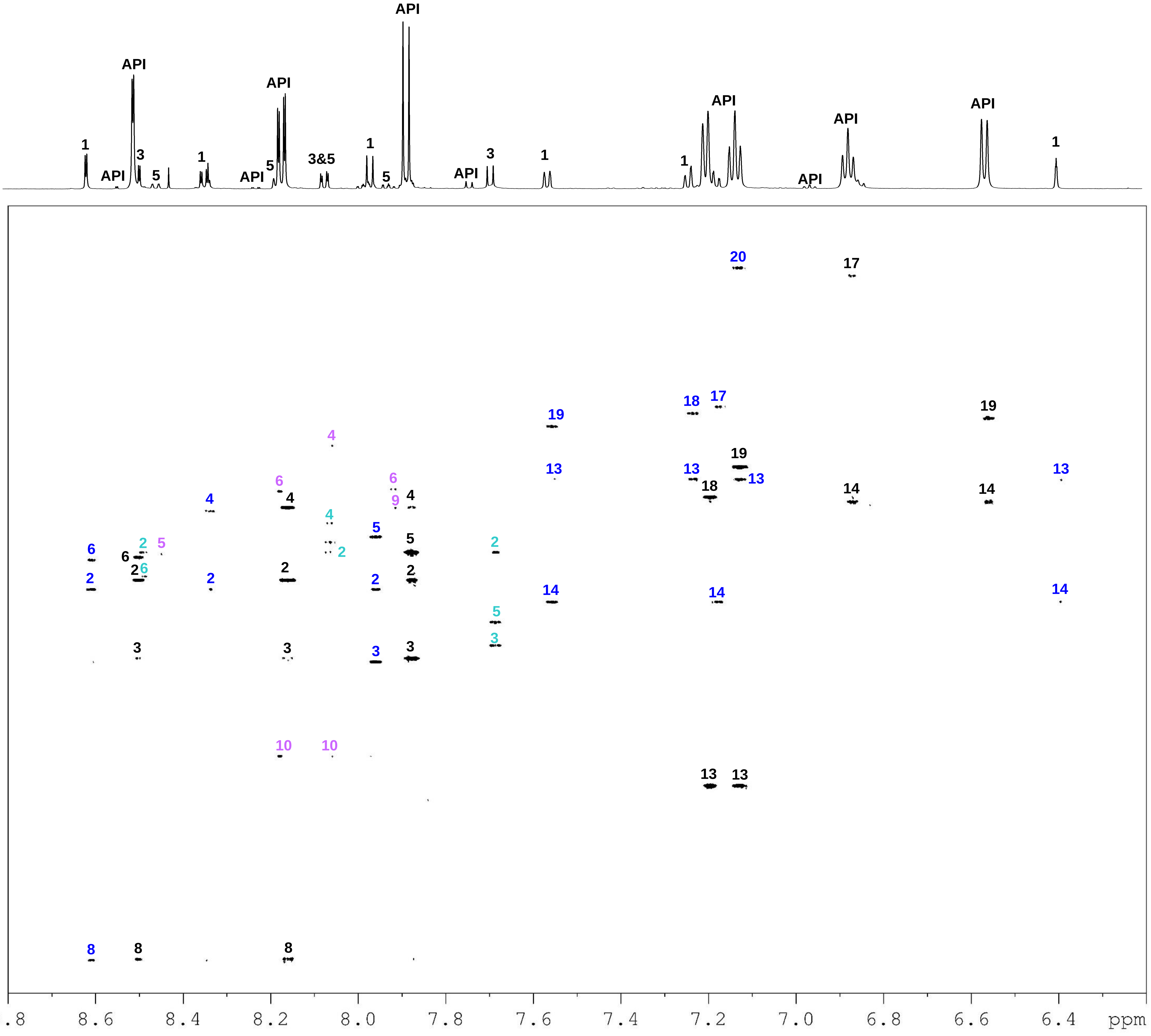

C)

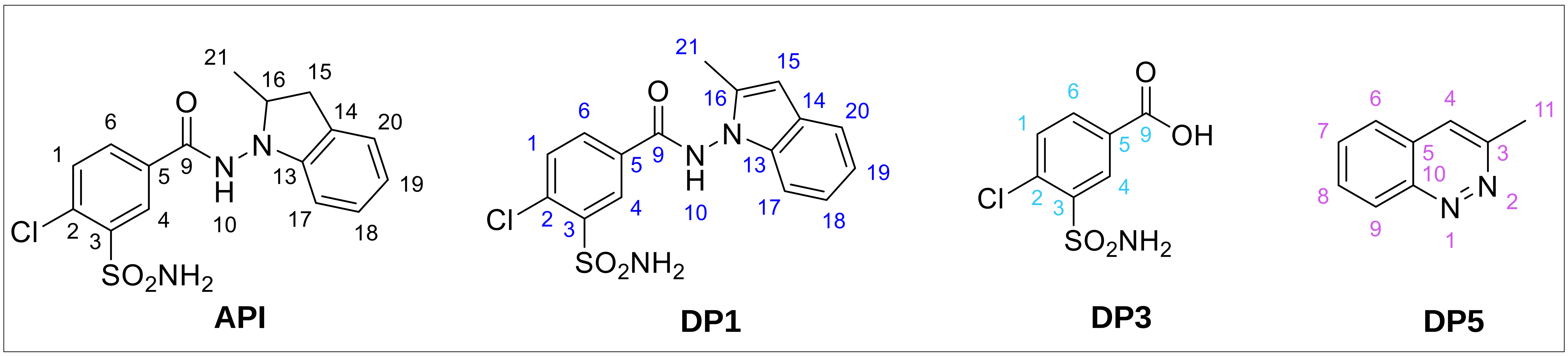

Figure S4. Complete NMR assignment of indapamide, DP1, DP3 and DP5 (labeled API, 1, 3 and 5) for HCl hydrolysis: A) Acquisition parameters; B) 2D HMBC spectrum with correlations ^{1}H-^{13}C of each compounds; C) Structures and numbering of each compounds.

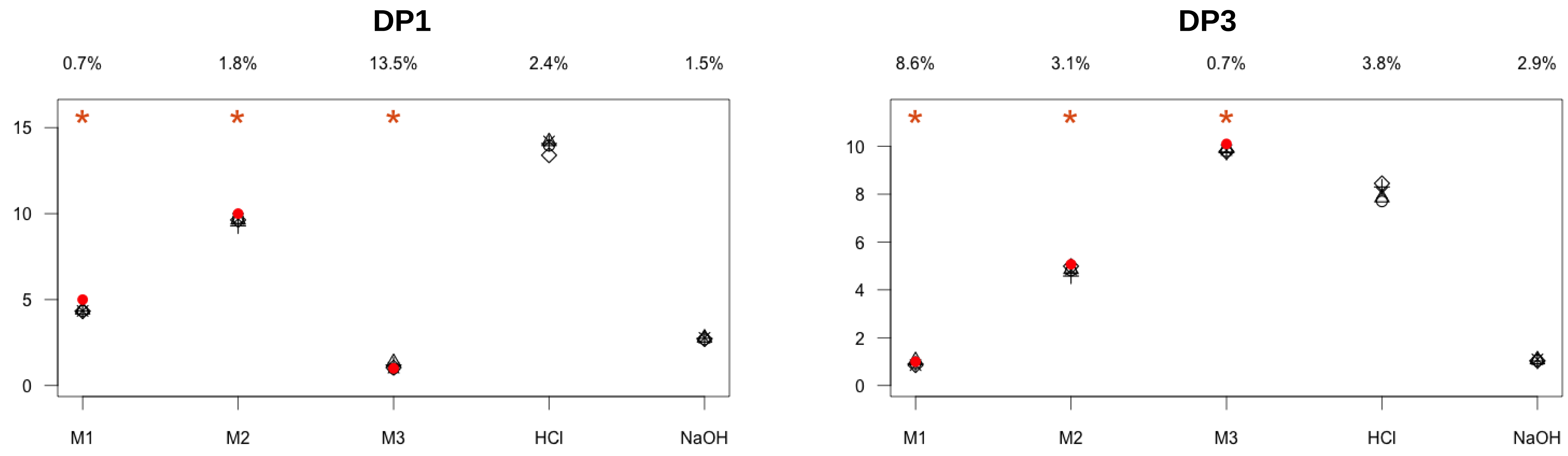

Figure S5. Quantification of DP1 and DP3 (in Y axis) in reconstitute mixtures and acid/alkaline degradations with qNMR methods for the pools (×, ◊, ○, +, Δ with ● represents the mean value). In the top of each figure, the coefficient of variation were calculated and the significant of result was described by * .
